# Estrogen receptor α dependent regulation of estrogen related receptor β and its role in cell cycle in breast cancer

**DOI:** 10.1186/s12885-018-4528-x

**Published:** 2018-05-30

**Authors:** B. Madhu Krishna, Sanjib Chaudhary, Dipti Ranjan Mishra, Sanoj K. Naik, S. Suklabaidya, A. K. Adhya, Sandip K. Mishra

**Affiliations:** 1 Cancer Biology Lab, Institute of Life Sciences, Nalco Square, Chandrasekharpur, Bhubaneswar, Odisha 751023 India; 2 Present address: Department of Biochemistry and Molecular Biology, University of Nebraska Medical Center (UNMC), Omaha, NE USA; 3 Department of Gene Function & Regulation, Institute of Life Sciences, Nalco square, Chandrasekharpur, Bhubaneswar, Odisha 751023 India; 4 Tumor Microenvironment and Animal Models Lab, Department of Translational Research and Technology Development, Institute of Life Sciences, Nalco square, Chandrasekharpur, Bhubaneswar, Odisha 751023 India; 5 Department of Pathology, Kalinga Institute of Medical Sciences, Chandaka Industrial Estate, KIIT Rd, Patia, Bhubaneswar, Odisha India

**Keywords:** Breast cancer, Estrogen receptor α (ERα), Estrogen related receptor β (ERRβ), Estrogen/17beta-estradiol (E2), Promoter, Tissue microarray (TMA), ChIP, Re-ChIP, Fluorescence-activated cell sorting analysis (FACS)

## Abstract

**Background:**

Breast cancer (BC) is highly heterogeneous with ~ 60–70% of estrogen receptor positive BC patient’s response to anti-hormone therapy. Estrogen receptors (ERs) play an important role in breast cancer progression and treatment. Estrogen related receptors (ERRs) are a group of nuclear receptors which belong to orphan nuclear receptors, which have sequence homology with ERs and share target genes. Here, we investigated the possible role and clinicopathological importance of ERRβ in breast cancer.

**Methods:**

Estrogen related receptor β (ERRβ) expression was examined using tissue microarray slides (TMA) of Breast Carcinoma patients with adjacent normal by immunohistochemistry and in breast cancer cell lines. In order to investigate whether ERRβ is a direct target of ERα, we investigated the expression of ERRβ in short hairpin ribonucleic acid knockdown of ERα breast cancer cells by western blot, qRT-PCR and RT-PCR. We further confirmed the binding of ERα by electrophoretic mobility shift assay (EMSA), chromatin immunoprecipitation (ChIP), Re-ChIP and luciferase assays. Fluorescence-activated cell sorting analysis (FACS) was performed to elucidate the role of ERRβ in cell cycle regulation. A Kaplan-Meier Survival analysis of GEO dataset was performed to correlate the expression of ERRβ with survival in breast cancer patients.

**Results:**

Tissue microarray (TMA) analysis showed that ERRβ is significantly down-regulated in breast carcinoma tissue samples compared to adjacent normal. ER + ve breast tumors and cell lines showed a significant expression of ERRβ compared to ER-ve tumors and cell lines. Estrogen treatment significantly induced the expression of ERRβ and it was ERα dependent. Mechanistic analyses indicate that ERα directly targets ERRβ through estrogen response element and ERRβ also mediates cell cycle regulation through p18, p21^cip^ and cyclin D1 in breast cancer cells. Our results also showed the up-regulation of ERRβ promoter activity in ectopically co-expressed ERα and ERRβ breast cancer cell lines. Fluorescence-activated cell sorting analysis (FACS) showed increased G0/G1 phase cell population in ERRβ overexpressed MCF7 cells. Furthermore, ERRβ expression was inversely correlated with overall survival in breast cancer. Collectively our results suggest cell cycle and tumor suppressor role of ERRβ in breast cancer cells which provide a potential avenue to target ERRβ signaling pathway in breast cancer.

**Conclusion:**

Our results indicate that ERRβ is a negative regulator of cell cycle and a possible tumor suppressor in breast cancer. ERRβ could be therapeutic target for the treatment of breast cancer.

## Background

Breast cancer (BC) is the second leading cause of deaths in women worldwide. The occurrence rate of male BC is rare; it is the most predominant cancer in women in United States (US) [1]. It has been estimated that 2,52,710 new cases and 40,610 deaths are expected in women during the year 2017 in U.S alone [2]. BC has been recently classified based on molecular patterns of gene expression into different subtypes [3]. Luminal subtype which is characterized by the presence of estrogen receptor (ER) comprises ~ 60–70% of BC and responds better to endocrine therapy i.e.; tamoxifen [4]. However, due to lack of therapy ER negative BC demands to identify molecular targets that might have therapeutic importance.

ERs are a group of nuclear receptors regulated by steroid hormone estrogen (E2). ERs are of three types; ERα, ERβ and ERγ [5]. In the presence of E2, ERs either as a homodimer or heterodimer bind to estrogen response elements (ERE) present in the target gene promoter to regulate its transcriptional activity [6–9]. ERα and ERβ expresses widely in different tissues including brain [10]. Although ERα causes cell migration, division, tumor growth in response to E2 [11, 12], ERβ inhibits migration, proliferation and invasion of breast cancer cells [13–15]. Besides being a key molecule in breast cancer pathogenesis, ERα plays an anti-inflammatory role in brain [16].

Estrogen related receptors (ERRs) are a group of nuclear receptors family having sequence homology with ERs and act as transcriptional regulators [17]. Unlike ERs, ERRs are lesser known affected by steroid hormone estrogen. Since a decade after discovery, no natural ligand has been found for these receptors, hence called as orphan nuclear receptors [18, 19]. Estrogen related receptors (ERRs) share target genes with ERs [20, 21]. Estrogen related receptors (ERRs) are also of 3 types; ERRα, ERRβ and ERRγ [22–25]. ERRs recognize a short sequence referred as ERR- responsive element (ERRE) on target gene promoter and regulate their transcriptional activity [26–29]. The distribution of ERRs varies, although ERRα expresses in various tissues such as kidney, skeletal muscle, intestinal tract etc, but ERRγ restrict themselves mainly in heart and kidney [30, 31]. ERRα mediates cell proliferation through pS2 [21] and plays an important role in regulation of mitochondrial metabolism in breast cancer cells [29, 32]. Knockdown of ERRα leads to cardiac arrest in mice [33]. ERRβ expresses in early stages of mouse embryonic development [34]. Mutation in ERRβ leads to autosomal recessive non syndromic hearing impairment in mice [35]. ERRβ acts as tumor suppressor in prostate cancer by up-regulating p21^cip^ [36]. Recent studies have demonstrated the abrogated expression of ERRβ in breast cancer cells [37]. In this study we have demonstrated that ERα regulates the expression of ERRβ through estrogen in breast cancer. We demonstrated the elevated levels of ERRβ in normal breast tissues and ER + ve breast tumors compared to breast carcinoma and ER-ve breast tumors respectively. We also demonstrated that ectopic expression of ERRβ causes significant up-regulation of p18 and p21^cip^ in breast cancer cells and also arrest cell cycle in G0/G1 phase. Thus our data, suggest the tumor suppressor role of ERRβ which provide therapeutic potential to ERRβ signaling pathway.

## Methods

### Tissue microarray

Breast cancer tissue microarray slides (Cat No. BR 243v, BR 246a) were purchased from US Biomax (Rockville, MD, USA). The slides were stained by anti-ERRβ antibody at 1:50 dilution (sc-68879, Santa Cruz, Dallas, TX, USA) and were further processed using ABC system (Vector Laboratories, Bulingame, CA, USA) as described previously [38]. The images were captured under Leica microscope (Wetzlar, Germany) using LAS EZ software version 2.1.0. The slides were examined and scoring was done by an experienced pathologist. The intensity score was calculated based on staining for ERRβ and was assigned from 0 to 3 (0 indicating no staining; 1+ weakly stained; 2+ moderately stained and 3+ strongly stained positively). The percentage of positively stained cells were scored as follows, 0- no positive staining; 1+, 1–25% positively stained cells; 2+, 26–50% positively stained cells; 3+, 51–70% positively stained cells; 4+, > 70% positively stained cells. The composite score was calculated using both intensity score and the percentage of positive cells as it is a product of both scores. The composite score range was given from 0 to 12. The samples scored < 3 were considered as low categorized; 3–5 moderately categorized; ≥ 6 highly categorized. The graph was plotted using composite scores using GraphPad Prism version 6.01.

### Cell culture and treatment

Human estrogen receptor positive breast cancer cell lines (MCF7 and T47D) and estrogen receptor negative breast cancer cell line (MDA-MB231) were purchased from cell repository of National Center for Cell Sciences (NCCS, Pune, India) and were cultured and maintained as described previously [39]. MCF10A was a kind gift from Dr. Annapoorni Rangarajan (IISc, Bangalore, India) was maintained as previously described [40]. For estrogen treatments, MCF7 and T47D cell lines were grown in phenol red free medium for 48 h prior to 17beta-estradiol (E2) (Sigma-Aldrich, St. Louis, MO, USA) treatment. MCF7 cells were treated with10 and 100 nM E2 concentrations for different time points 0, 6, 12, 24, 48 h. For inhibition studies, MCF7 cells were treated with 1 μM of tamoxifen (Sigma-Aldrich) [41], 10 nM E2 individually and in combination with both for 24 h prior to harvesting of cells. MCF7 cells were transfected with ERα shRNA (SHCLND-NM_000125, Sigma-Aldrich) and were culture and maintained for 48 h prior to further experiments.

### Cloning of 5′ flanking region of *ERRβ* gene

Genomic DNA was isolated from MCF7 cells as per the standard protocol [42]. A 1014 bp genomic fragment of the ERRβ gene, from − 988 to + 26 bp relative to the start sequence of exon1 (designated as + 1) was amplified by PCR using 50–100 nanograms of genomic DNA as a template. The genomic fragment was amplified with *KpnI* and *XhoI* restriction sites using primer sequences provided in Table 1. The parameters of PCR reaction were as follows: initial denaturation 95 °C for 5 min, 35 cycles of 95 °C for 30 s, 56 °C for 30 s, 72 °C for 1 min and a final extension of 72 °C for 10 min. The amplified samples were resolved in 0.8% (*w*/*v*) agarose gel and purified using Gene elute gel extraction kit (Sigma-Aldrich) according to manufacturer’s protocol. Both the purified PCR product and PGL3 basic luciferase vector were digested using *KpnI* and *XhoI* (Thermo Scientific, Waltham, MA, USA) restriction enzymes for 4 h at 37 °C and purified. The restriction digested PCR product and PGL3 vectors were ligated using T4 DNA ligase (New England BioLabs, Inc., Ipswich, MA, USA) and clone was confirmed by sequencing and designated as pGL3*-ERRβ*.

**Table 1:** List of primers

S.No	Oligos	Sequence (5′-3′)
1	ERRβ Promoter F	ACAGGTACCTTGTACTCCAGTCTGGGCGA
2	ERRβ Promoter R	ACACTCGAGATGTCCCTGACCACACCTCT
3	RT-ERα F	AGCTCCTCCTCATCCTCTCC
4	RT-ERα R	TCTCCAGCAGCAGGTCATAG
5	RT-ERRβ F	CTATGACGACAAGCTGGTGT
6	RT-ERRβ R	CCTCGATGTACATGGAATCG
7	RT-p21^cip^ F	GAGGCCGGGATGAGTTGGGAGGAG
8	RT-p21^cip^ R	CAGCCGGCGTTTGGAGTGGTAGAA
9	RT-GAPDH F	AAGATCATCAGCAATGCCTC
10	RT-GAPDH R	CTCTTCCTCTTGTGCTCTTG
11	ERRβ EMSA Site 1F	GGACAAAAATAAGGTCAAGTTTCTTTGTTA
12	ERRβ EMSA Site 1R	TAACAAAGAAACTTGACCTTATTTTTGTCC
13	ERRβ EMSA Site 2F	ATTTAATGAGACAGGTCATTCATTCAGTCA
14	ERRβ EMSA Site 2R	TGACTGAATGAATGAATGACCTGTCTCATTAAAT
15	ERRβ chip ERE Site 1F	CCAGTCTGGGCGACAAGAGTGAAACTC
16	ERRβ chip ERE Site 1R	CCATTACAGTGGATTGTGGAG
17	ERRβ chip ERE Site 2F	CTCCACAATCCACTGTAATGG
18	ERRβ chip ERE Site 1R	CCAACTACCAGGAGAATAGGAGCAC

### Total RNA isolation and real-time PCR

Total RNA was isolated from MCF7, T47D, MDA MB-231 and ERα KD cells using Tri reagent (Sigma-Aldrich). A total of 500 ng was digested with DN*ase*-I enzyme (Sigma-Aldrich) and was subjected to cDNA synthesis using superscript II first strand synthesis kit (Thermo Scientific). Reverse transcription PCR and Quantitative reverse transcription PCR was performed using primers provided in Table 1. *GAPDH* was taken as an internal control and ΔΔCT values were calculated for Quantitative reverse transcription PCR. The Quantitative reverse transcription PCR results were plotted using GraphPad Prism version 6.01.

### Preparation of cell extracts and western blotting

The whole cell lysates from breast cancer cell lines (MCF10A, MCF7, T47D, MDA MB-231) were prepared using RIPA buffer (500 mM NaCl, 5 mM MgCl_2_, 1% Na deoxycholate, 20 mM Tris-HCl (pH 8.0), 10% glycerol, 1 mM EDTA, 100 mM EGTA, 0.1% NP40, 1% Triton X-100, 0.1 M Na_3_VO_4_, 1X Protease inhibitor). Approximately 20–40 microgram of protein was separated using 10–12% SDS-polyacrylamide gel and transferred onto PVDF membrane (GE Healthcare Life Sciences, Chalfont, UK). Blots were incubated with 5% nonfat milk for blocking and were further incubated with 1 μg each of subsequent antibodies ERα (8644, Cell signaling technology, Danvers, MA, USA), ERRβ (Sc-68879, Santa Cruz) [37], α-tubulin (Sigma-Aldrich), cyclin D1 (2978, Cell Signaling Technology), p21^cip^ (2947, Cell Signaling Technology), p18 (2896, Cell Signaling Technology) followed by corresponding HRP labeled secondary antibody. The blot was incubated with ECL (Santa Cruz) for 5 min and visualized in Chemidoc XRS+ molecular 228 imager (Bio-Rad, Hercules, CA, USA). α-tubulin was considered as a loading control. The western blot images were quantified using Image J software (NIH, Bethesda, MD, USA).

### Electrophoretic mobility shift assay

The nuclear fractions were isolated as described previously [41] using CelLytic NuCLEAR Extraction Kit (Sigma-Aldrich) and were stored at -80 °C for further use. In-vitro DNA-protein interaction was carried out using Electrophoretic mobility shift assay (EMSA). The oligonucleotide sequences having ERE site present in the ERRβ promoter region were synthesized and were designated as ERRβ EMSA site 1 (− 888 to − 859) and ERRβ EMSA site 2 (− 822 to − 793). The forward strands of both EMSA site 1 and EMSA site 2 were labeled at 5′ end with [γ^− 32^ P] ATP (BRIT, Hyderabad, India) using T4 polynucleotide kinase (Promega, Madison, USA). The 5′ labeled oligonucleotides were annealed with unlabeled reverse complementary strands incubating in annealing buffer (1 M Tris-HCl (pH 7.5), 4 M NaCl, 0.5 M MgCl_2_). The annealed oligonucleotides were incubated with nuclear extract for 20 min at RT in binding buffer [1 M Tris-HCl (pH 7.5), 50% (*v*/v) glycerol, 0.5 M EDTA, 1 M DTT, 50 mg/ml BSA, 4 M NaCl]. Poly(dI-dC) was used as a nonspecific competitor. For specific competition 100–150 fold excess unlabeled ERα consensus oligonucleotides were added to the reaction 10 min prior to adding 0.2 pmoles radiolabeled oligonucleotides. The DNA-protein complexes were separated in 6% polyacrylamide gel at 180 V for 1 h in 0.5X Tris-HCl/Borate/EDTA running buffer [40 mM Tris-Cl (pH 8.3), 45 mM boric acid and 1 mM EDTA] and was dried and autoradiographed.

### Chromatin immunoprecipitation assay (ChIP)

Chromatin immunoprecipitation was performed as prescribed previously with minor modifications [43]. MCF7 and T47D cells were grown in phenol red free DMEM, RPMI-1640 (PAN Biotech GmbH, Aidenbach, Germany) medium respectively, supplemented with 10% (*v*/v) charcoal treated FBS (PAN Biotech GmbH) for 48 h prior to E2 treatment. Cells were treated with 100 nM E2 for 48 h, fixed with 1% (v/v) formaldehyde and were washed twice with 1X PBS (10 mM PO_4_^3−^, 137 mM NaCl and 2.7 mM KCl). Cells were lysed in SDS lysis buffer (1% (*w*/*v*) SDS, 10 mM EDTA, 50 mM Tris-HCl (pH 8.1)) with protease inhibitor cocktail (Sigma-Aldrich) and were sonicated using Bioruptor ultrasonicator device (Diagenode S.A., Seraing, Belgium) at M2 amplitude strength. The sonicated samples were subjected to pre-clearing with protein A/G agarose beads (GE Healthcare Life Sciences). These pre-cleared samples were diluted with ChIP dilution buffer (0.01% (*w*/*v*) SDS, 1.1% (*v*/v) Triton X-100, 1.2 mM EDTA, 16.7 mM Tris-HCl (pH 8.1), 167 mM NaCl) and divided into two equal parts IgG and IP, 50 μl was taken as input and was stored at -80 °C. The IgG and IP were incubated with 1 μg of anti-IgG (Diagenode), anti-ERα (8644 s; Cell Signaling Technology) and anti-ERRβ (sc-68879, Santa Cruz) antibodies respectively. The protein-antibody complex was extracted by incubating the samples with protein A/G agarose beads. The protein-antibody-bead complex was extracted, washed with series of different washing buffers i.e. Low salt buffer [0.1% (v/v) SDS, 2 mM EDTA, 1% (v/v) Triton X-100, 20 mM Tris-HCl (pH 8.1) and 150 mM NaCl], High salt buffer [0.1% (v/v) SDS, 1% (v/v) Triton X-100, 2 mM EDTA, 20 mM Tris-HCl (pH 8.1) and 500 mM NaCl], LiCl salt buffer [0.25 M LiCl, 1% (v/v) NP-40, 1% (*w*/*v*) deoxycholic acid (sodium salt), 1 mM EDTA and 10 mM Tris-HCl (pH 8.1)], 1X TE [10 mM Tris-HCl (pH 8.1) and 1 mM EDTA] and were eluted using elution buffer (1% (*v*/*v*) SDS, 0.1 M NaHCO_3_). The eluted samples and input were reverse crosslinked with 5 M NaCl for 6 h at 65 °C followed by incubation with 0.5 M EDTA, 1 M Tris-HCl (pH 6.5) and proteinase K at 45 °C for 1 h. ChIP elutes were purified using phenol/chloroform and ethanol precipitated. DNA samples were further used to perform PCR analyses to confirm the binding of ERα and ERRβ on *ERRβ* promoter. The primer sequences used for ChIP PCR were provided in Table 1.

### Re-ChIP

Re-ChIP was performed as described previously with brief modifications [44]. The sonicated samples were incubated with 1 μg of anti-IgG (kch-504-250; Diagenode) and anti-ERα (8644 s; Cell Signaling Technology) antibodies. The antibody and protein complex was extracted using protein A/G agarose beads (GE Healthcare Life Sciences), washed with Re-ChIP wash buffer (2 mM EDTA, 500 mM NaCl, 0.1% (v/v) SDS, 1% (*v*/v) NP40) and eluted with Re-ChIP elution buffer (1X TE, 2% SDS, 15 mM DTT). The eluted samples were further subjected to secondary immunoprecipitation with 1 μg of anti-ERRβ (Sc-68879, Santa Cruz) primary antibody. The complex was extracted using protein A/G agarose beads (GE Healthcare Life Sciences), washed with different buffers (Low salt buffer, High salt buffer, LiCl salt buffer, 1X TE) and eluted. The eluted samples were further subjected to reverse crosslinking followed by phenol/chloroform/isoamyl alcohol DNA isolation. The DNA samples were further used to perform PCR to confirm the binding of ERα and ERRβ complex on the *ERRβ* promoter.

### Transfection and luciferase assay

MCF7 cells were grown in 24 well plates in phenol red free DMEM supplemented with 10% (v/v) charcoal treated fetal bovine serum 48 h prior to estrogen (E2) treatment. Cells were transfected with pGL3-*ERR*β, pEGFP-*ERα* [41], pEYFP C1-ERRβ [37], pRL-Renilla luciferase construct (Promega) in different combinations using jetPRIME-polyplus-transfection reagent (Polyplus transfection, New York, NY, USA) according to manufacture protocol. Post 24 h transfection cells were treated with 100 nM E2 and vehicle and were allowed to grow for 24 h. Luciferase assay was performed using Dual luciferase assay detection kit (Promega) according to manufacture protocol. Luciferase readings were obtained and were normalized with Renilla luciferase activity. The graph was plotted with normalized readings using GraphPad Prism software version 6.01.

### Fluorescence-activated cell sorting analyses (FACS) for cell cycle

MCF7 Cells (3 × 10^5^) were grown in 6 well plates in Dulbecco’s Modified Eagle Medium supplemented with 10% charcoal treated fetal bovine serum at 37 °C for 24 h prior to transfection with pEYFP C1-ERRβ construct and were allowed to grow for 48 h. Cells were further harvested and were treated with 70% ethanol for fixation, washed with ice cold 1X PBS thrice and were stained with DNA stain propidium iodide (PI) at 37 °C. Sorting was performed and were analyzed using BD LSRFortessa (BD Biosciences) as described previously [45].

### Statistical analysis

The statistical significance was analyzed using unpaired t-test for 2-group comparison. Each data represents the mean ± SEM from three independent experiments. *P*-value < 0.05 was considered as statistically significant. One-way ANOVA test was performed to analyze the statistical significance of multiple group comparison. *P*-value < 0.05 was considered as statistically significant and were represented in respected figures accordingly.

## Results

### Decreased expression of ERRβ in breast carcinoma

**Fig. 1:**
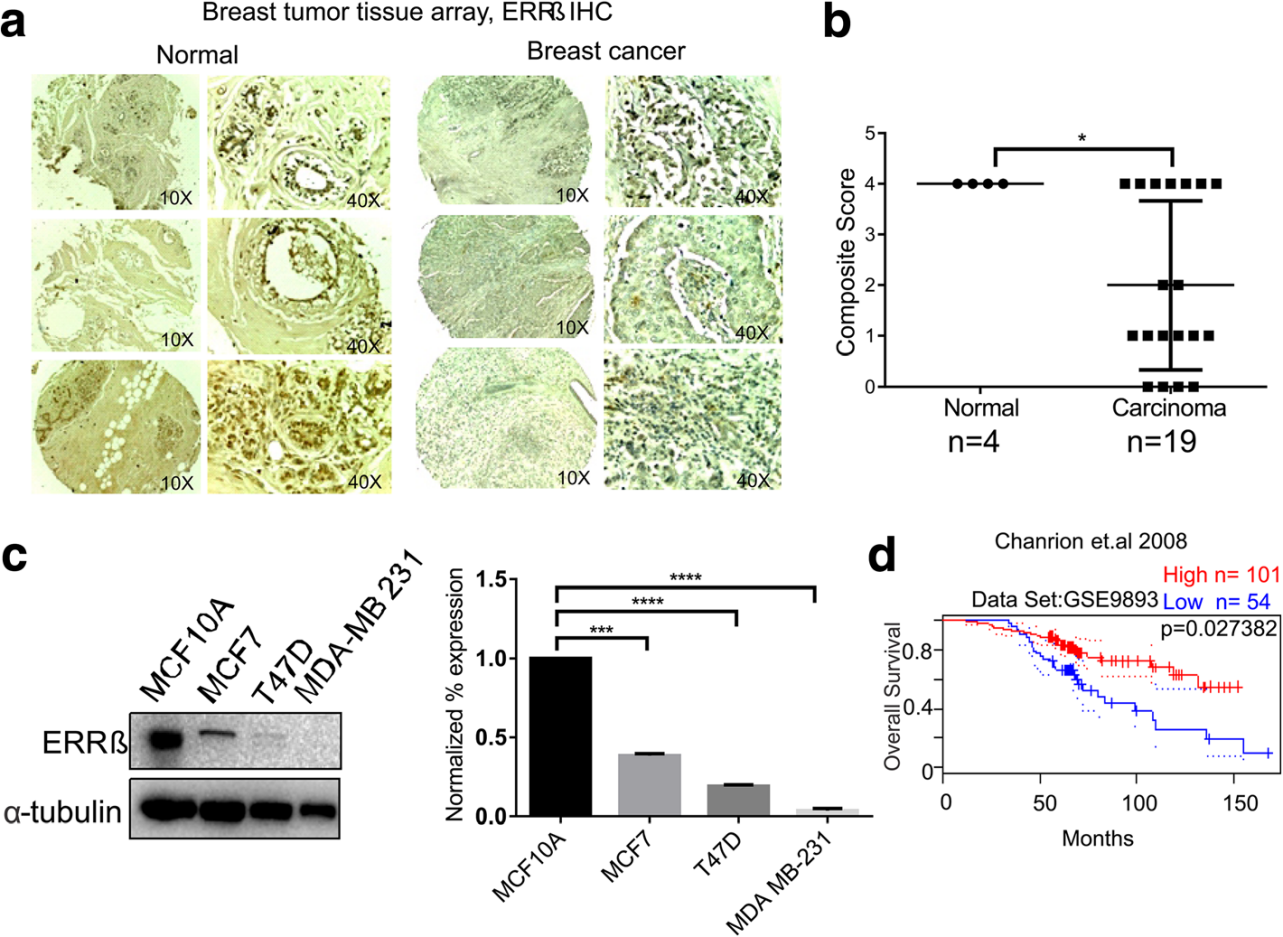
Expression of ERRβ in normal vs breast cancer tumor samples, cell lines and its pathological significance. **a** Immunohistochemical staining of tissue microarray slides using ERRβ antibody in both normal (*n* = 4) and breast carcinoma tissues (*n* = 19). Increased expression of ERRβ in normal tissues compared with breast carcinoma. **b** Graphical representation of IHC composite score of each tissue microarray sample. Composite score was calculated for each sample using both intensity score and percentage of cells positive for ERRβ staining (composite score < 3 low categorized; 3–5 moderately categorized; ≥ 6 highly categorized). Graph was plotted using composite score and *p*-values were calculated using 2-group t-test (*p* < 0.05 considered as significant). **c** Western blots revealing high expression of ERRβ in normal breast cell line (MCF10A), than breast cancer cell lines (MCF7, T47D, and MDA-MB-231). Densitometry analyses of ERRβ expression in normal and breast cancer cell lines, One-way ANOVA test was performed to acquire statistical significance (*p < 0.05, **p < 0.01, ***p < 0.001, ****p < 0.0001). **d** Kaplan-Meier survival curve of Chanrion et al. (Dataset: GSE9893) correlated higher expression of ERRβ with favorable survival (*p* = 0.027382)

The role of ERRβ in breast carcinoma has not been much elucidated with few reports published recently [37, 46]. To determine the role of ERRβ expression in breast carcinogenesis, we performed immunohistochemistry (IHC) using commercially available tissue microarray slides (TMA) purchased from US Biomax (https://www.biomax.us/) which consist of 24 samples consisting of both breast carcinoma and adjacent normal breast tissue samples. Among the 24 samples, 4 (16.66%) were negative and 19 (79.11%) were positive for ERRβ staining and 1 sample was stromal tissue. Our IHC staining (composite score) showed a significant decreased expression of ERRβ in breast carcinoma tissues compared to adjacent normal breast tissues (Fig. 1a and b). We next performed western blot (WB) analyses of whole cell lysates isolated from breast cancer cells and immortalized normal breast cells. WB analyses indicated significantly low levels of ERRβ expression in breast cancer cell lines compared to immortalized breast cell line, MCF10A (Fig. 1c). The publicly available dataset, GEO accession: GSE9893 was screened and analyzed for ERRβ expression and survival of breast cancer patients. As Kaplan-Meier survival analyses showed a significant overall survival in patients with high ERRβ expression (*p* = 0.027382) suggesting the anti-tumorigenic role in breast cancer (Fig. 1d) [47]. Thus our results indicate that ERRβ expression is decreased in breast carcinoma patients, breast cancer cell lines and also has pathological implications in breast cancer.

### ERRβ expression is ERα dependent

**Fig. 2:**
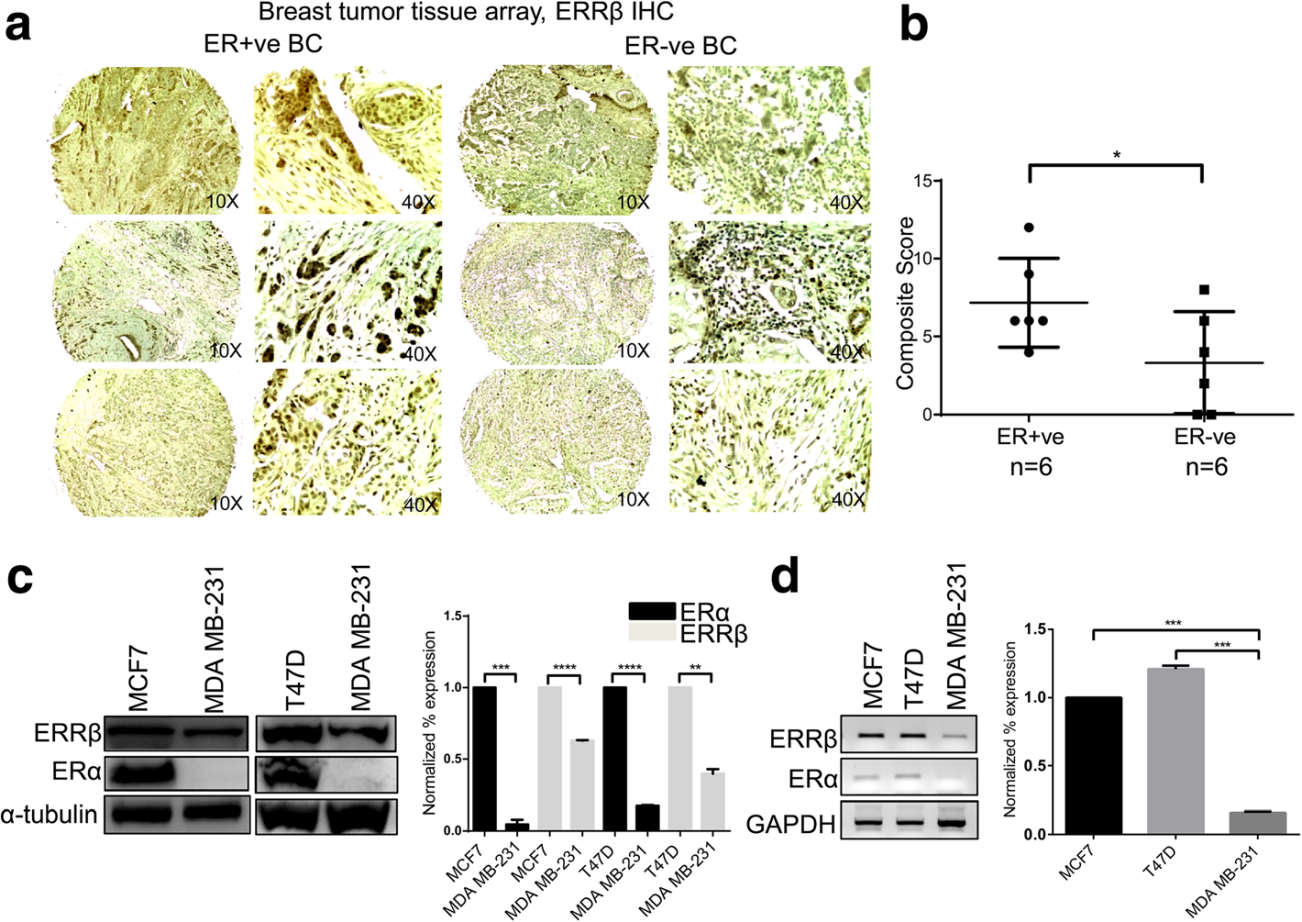
Correlation of ERRβ expression with ERα in breast tumors and cell lines. **a** Immunohistochemical staining with ERRβ antibody in ER + ve and ER-ve breast cancer patients. Elevated expression of ERRβ was found in ER + ve (*n* = 6) compared to ER-ve (*n* = 6) breast cancer patient samples. **b** Graphical representation of IHC composite scores of each tissue microarray sample showing significant elevated expression of ERRβ in ER + ve than in ER-ve breast cancer patient samples. Graph was plotted using composite score and *p*-values were calculated using 2-group t-test (*p* **<** 0.05 considered as significant). **c**, **d** Western blots, Reverse transcription polymerase chain reaction (RT PCR) and densitometry analysis results representing elevated levels of ERRβ in ER + ve breast cancer cells (*p < 0.05, **p < 0.01, ***p < 0.001, ****p < 0.0001). Statistical significance for relative gene expression (RT PCR) and normalized percentage of expression (WB) was analyzed using One-way ANOVA and unpaired t-test respectively (*p*-value < 0.05 was considered as significant)

**Fig. 3:**
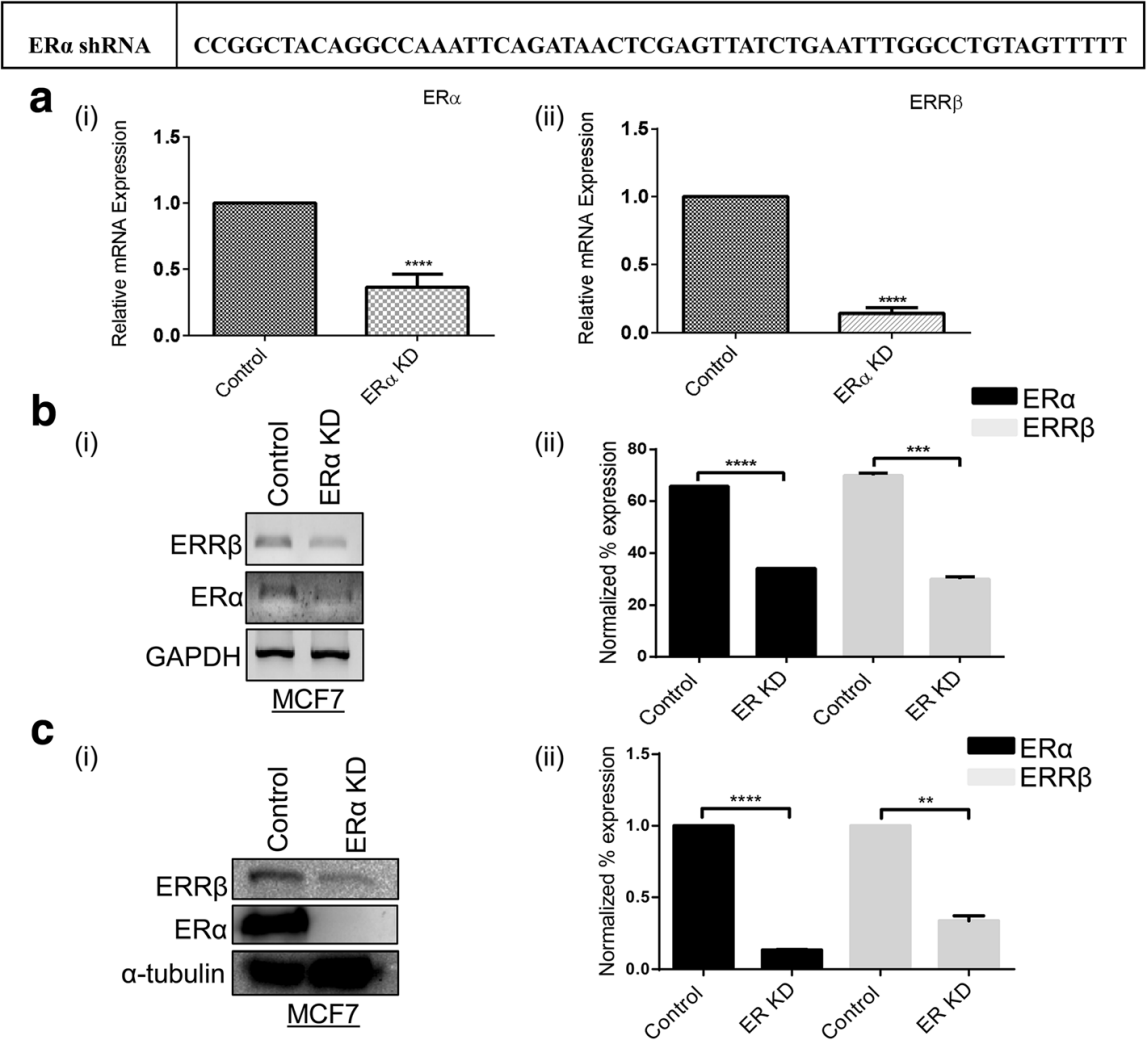
Expression of ERRβ is ERα dependent. Efficient knockdown of ERα showing significant decrease in the expression of ERRβ in MCF7 cells. **a**, **b** Quantitative Real-time PCR (qRT-PCR) and Reverse transcription polymerase chain reaction (RT-PCR) results showing decreased expression of ERRβ in ERα depleted MCF7 cells. Housekeeping gene *GAPDH* treated as control and ΔCt, ΔΔCt, 2^-ΔΔCt^ values were calculated and graph was plotted using 2^-ΔΔCt^ values. Fold change ≥ 2 was considered as significant. *p*-values were calculated using 2-group t-test (*p < 0.05, **p < 0.01, ***p < 0.001, ****p < 0.0001). **c** Western blot revealing the depleted expression of ERRβ in ERα Knockdown MCF7 (ERα KD) cells

To define the role of ERRβ in breast carcinogenesis, we elucidated the expression of ERRβ in ER + ve and ER-ve breast cancer patients in tissue microarray slides (TMA). The breast cancer TMA slide consist of 24 samples with both ER + ve and ER-ve breast carcinoma and adjacent normal breast tissues. IHC showed 2 (8.33%) samples that were negatively stained while 22 (91.67%) samples were positively stained for ERRβ expression. Interestingly, we found that composite score for ERRβ IHC staining was significantly high in ER + ve breast cancer patients (*n* = 6) than in patients with ER-ve receptor status (*n* = 6) suggesting that ERRβ expression might be controlled by ERα (Fig. 2a, b). To further confirm this observation we performed western blot and reverse transcription PCR (RT-PCR) for ERRβ in ER + ve (MCF7 and T47D) and ER-ve (MDA-MB231) breast cancer cell lines and the expression of ERRβ was found to be ERα dependent (Fig. 2c, d). We further confirmed these findings through short hairpin ribonucleic acid (shRNA) knockdown of ERα in MCF7 cells. We found that depletion of ERα by knockdown showed a significant decrease of ERRβ expression in MCF7 cells (Fig. 3a (i & ii), b (i & ii) and c (i & ii)). These results suggest for the first time that expression of the orphan receptor ERRβ is ERα status dependent and may have clinical significance in breast cancer pathogenesis.

### Estrogen dependent expression of ERRβ in ER + ve breast cancer cells

**Fig. 4:**
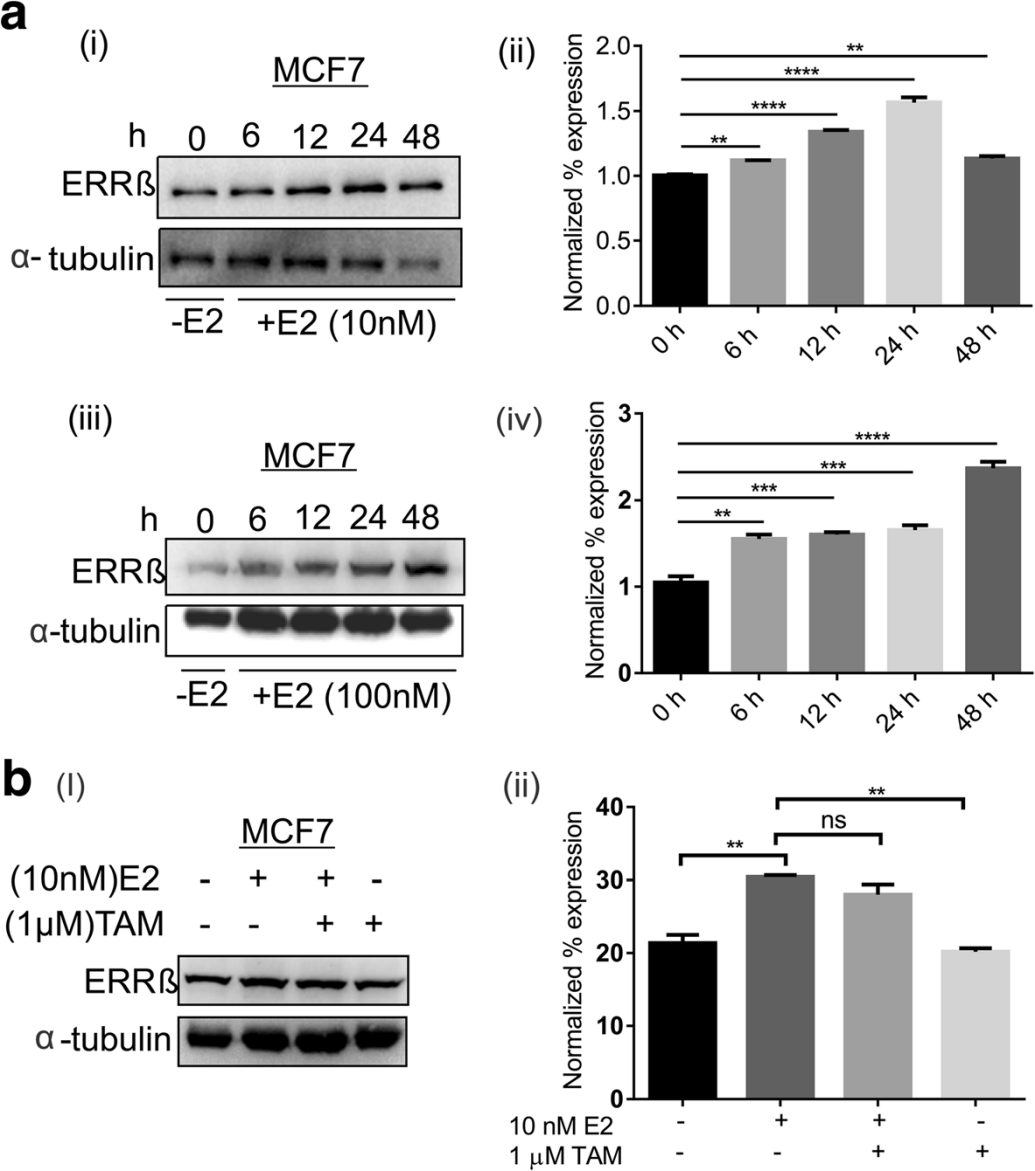
Estrogen regulates the expression of ERRβ. **a** Western blots and densitometry analyses showing up-regulation of ERRβ upon estrogen treatment at different concentrations [10 nM (i) & 100 nM (ii)] for different time points (0, 6, 12, 24, 48 h) in MCF7 cells. MCF7 cells showed > 2 fold high expression of ERRβ upon the treatment of 100 nM E2 treatment. **b** Combinatorial treatment of MCF7 cells with estrogen and tamoxifen decrease ERRβ expression. The association between normalized percentage expression in different groups were analyzed using One-way ANOVA test (ns- no significance, *p ≤ 0.05, **p ≤ 0.01, ***p ≤ 0.001, ****p ≤ 0.0001)

As we have shown the correlation between ERα and ERRβ in ER + ve patient samples and breast cancer cells, we therefore analyzed the effect of estrogen on ERRβ expression. MCF7 cells were treated with estrogen (10 & 100 nM) for different time intervals (0, 6, 12, 24 & 48 h) and western blot was performed. A significant increase in the expression of ERRβ (> 2 fold) was observed with estrogen treatment (100 nM) (Fig. 4a (iii & iv)) and effect of estrogen was observed at time point as low as 6 h with highest expression (~ 5 fold) at 48 h. It is to be noted that the treatment with lower concentration of estrogen (10 nM) also showed significant change in MCF7 cells after 12 to 24 h (Fig. 4a (i & ii)). However the estrogen mediated ERRβ up-regulation was inhibited with tamoxifen treatment (Fig. 4b). These results suggest that ERRβ expression in ER + ve breast cancer cells is estrogen dependent.

### ERα regulates ERRβ by binding to ERE sites present in the 5′ flanking region of *ERRβ*

**Fig. 5:**
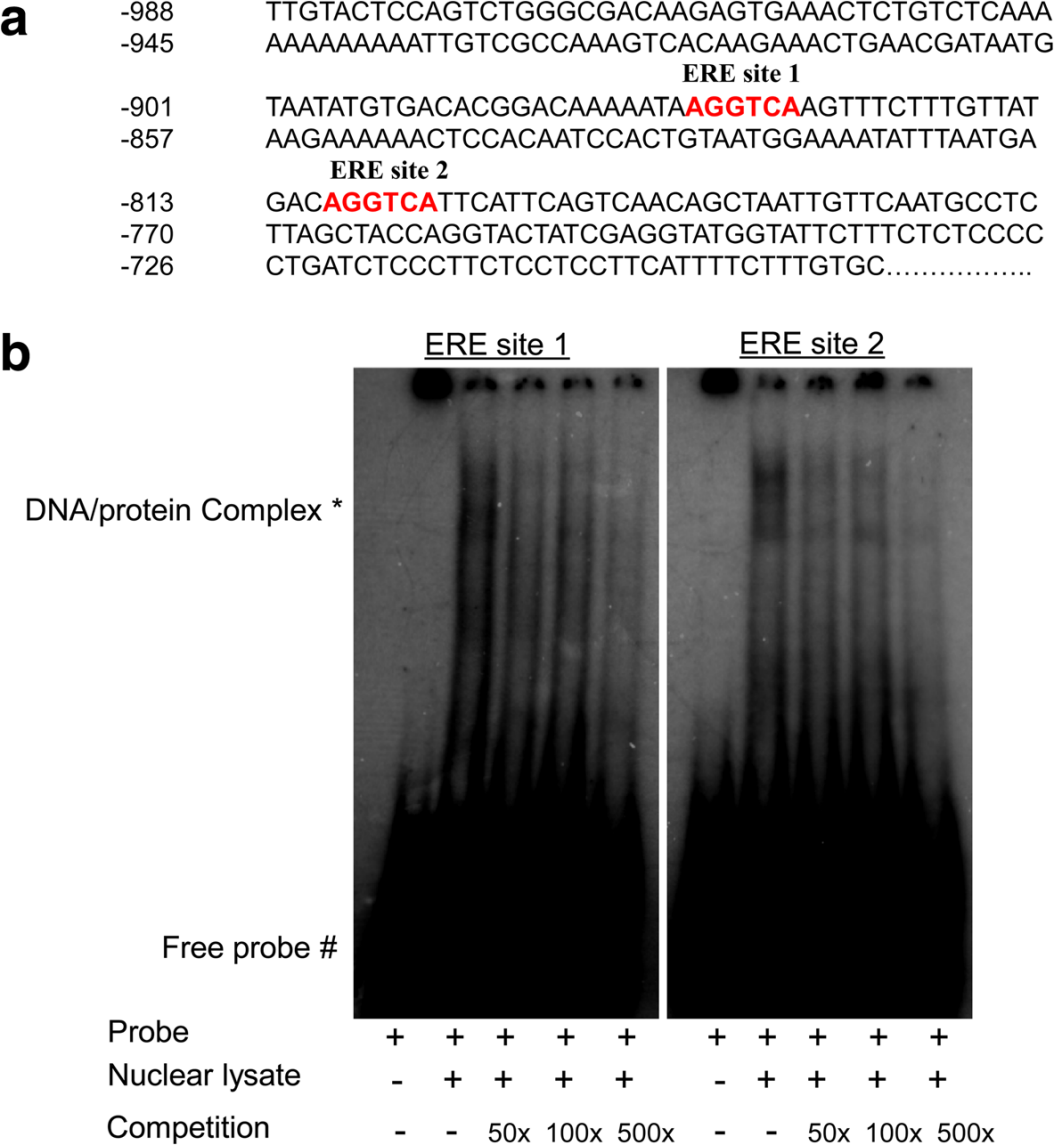
ERα interacts to *ERRβ* promoter in-vitro*.* **a** Schematic representation of two functional half ERE sites present in *ERRβ* promoter. Half ERE sites were situated from − 877 to − 872 and − 810 to − 805 respectively in the upstream region of *ERRβ* promoter. **b** Electrophoretic mobility shift assay (EMSA) representing the binding of ERα on both the half ERE sites in *ERRβ* promoter region. Oligonucleotides including half ERE site were labeled with [γ^− 32^ P] ATP and were incubated for 20 min with nuclear lysate extracted from MCF7 cells. An unlabeled ERE consensus oligonucleotide sequences were used as cold probe for competition at 50, 100 and 500 folds molar excess. Oligonucleotides were separated in 6% polyacrylamide gel using 0.5X TBE (Tris/Borate/Ethylenediaminetetraacetic acid) for 1 h at 180 V. The gel was dried and was autoradiographed

**Fig. 6:**
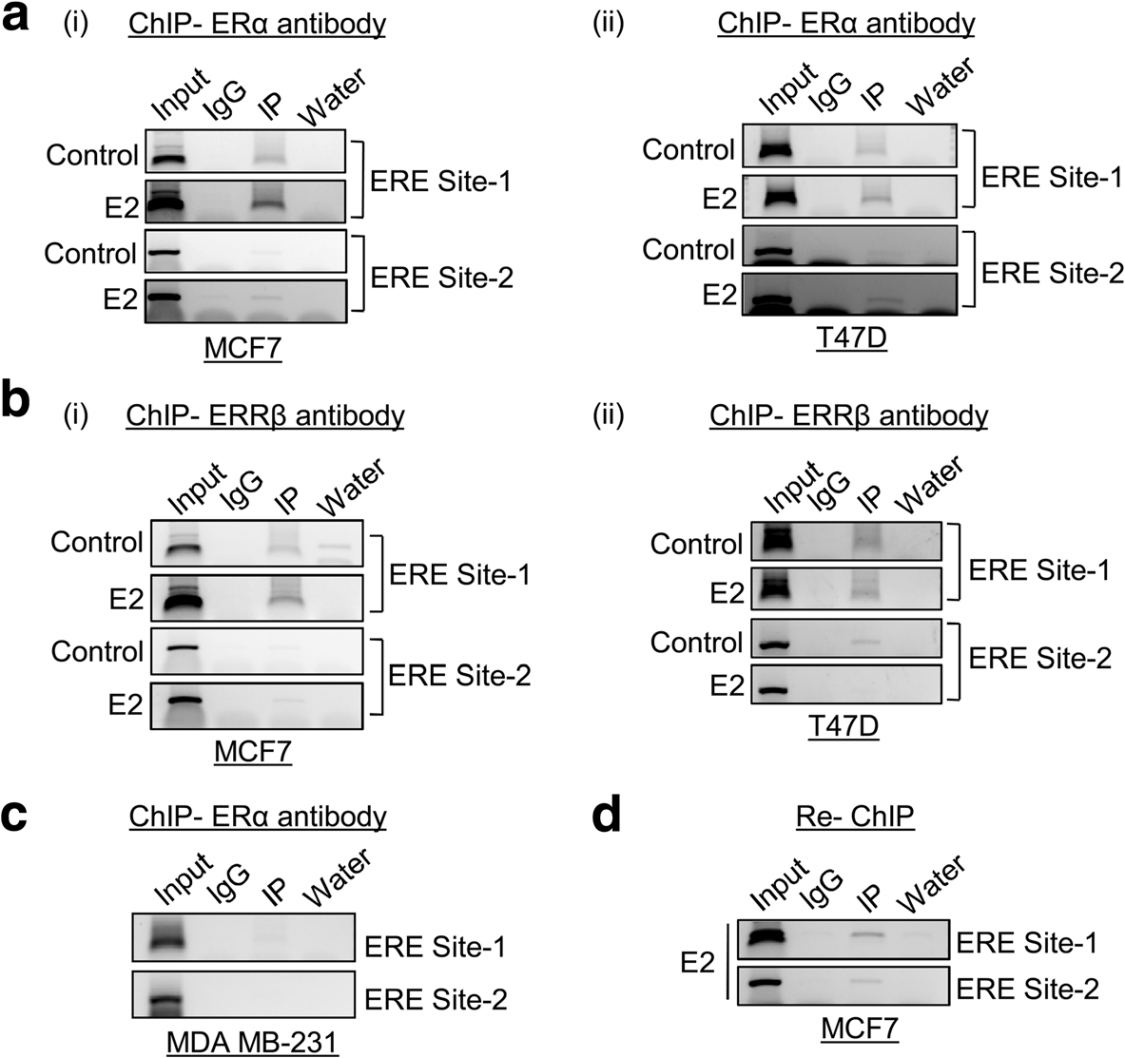
Estrogen facilitates binding of ERRβ on half EREs through ERα. **a** ChIP assay showing ERα binding to half EREs on *ERRβ* promoter in-vivo in the presence of estrogen in (i) MCF7 and (ii) T47D cells. **b** ERRβ binding in the upstream region of *ERRβ* promoter upon estrogen treatment in (i) MCF7 and (ii) T47D cells. **c** ER-ve breast cancer cells (MDA-MB 231) showing no binding of ERα on *ERRβ* promoter and used as a negative control. **d** Re-ChIP shows binding of ERα and ERRβ heterodimer complex on half ERE sites present on *ERRβ* promoter

To understand the role of estrogen receptor α in the regulation of *ERRβ*, the 5′ flanking sequence of *ERRβ* was screened for the presence of ERE sites manually. Two putative half estrogen responsive elements (ERE sites) were found and designated as ERE site1 (− 877 to − 872) and ERE site 2 (− 810 to − 805) (Fig. 5a). To confirm the binding of ERα to the putative half ERE sites present in the 5′ flanking sequence of ERRβ, electrophoretic mobility shift assay (EMSA) was performed. The oligonucleotides designated as ERRβ EMSA site1 and ERRβ EMSA site2 were radio labeled with [γ^− 32^ P] ATP and incubated with nuclear extracts isolated from MCF7 cells. EMSA clearly shows that ERα can bind to both the putative sites (ERE site 1 and ERE site 2). The specificity of the protein bound to the sites was further confirmed by competing with 50–500 fold molar excess of unlabelled estrogen response element (ERE) consensus sequence. The unlabelled ERE consensus completely abolish the DNA/protein complex suggesting the binding of ERα (Fig. 5b). Further chromatin immunoprecipitation assay (ChIP) was carried out to confirm the binding of ERα on ERRβ promoter in-vivo. MCF7 and T47D cells were treated with estrogen for 48 h and were subjected to ChIP procedure using ERα monoclonal antibody. The isolated immunoprecipitated DNA fragments were then subjected to PCR amplification. The ChIP PCR suggests the enriched binding of ERα on both the half ERE sites present on the 5′ flanking region of *ERRβ* during estrogen stimulation compared with the untreated samples and binding of ERα on ERE site 1 is stronger than ERE site 2 (Fig. 6a (i) and (ii)). However we did not observe any binding of ERα in the same sites in MDA-MB231 cell line as expected and used as a negative control during the ERα ChIP procedure. (Fig. 6c). Apart from ERα recruitment to ERE elements, ERRβ may also be co-recruited on its own promoter through ERα. Previous reports have already proved that estrogen treatment lead to formation of heterodimer between ERα and ERRβ proteins [48]. Our previous results also suggest the increased nuclear localization of ERα in the presence of estrogen. Therefore we hypothesize that the binding of ERRβ on its own promoter may be through ERα in the form of heterodimer. To test this hypothesis we initially performed in-vivo ChIP assay using ERRβ specific antibody and found that ERRβ binds to the half ERE sites present on its 5′ flanking region in the presence of estrogen (Fig. 6b (i) and (ii)). We then performed Re-ChIP in which both the ERα and ERRβ antibodies were used. Re-ChIP PCR clearly showed that ERα along with ERRβ binds to the half ERE sites present in the 5′ flanking region of ERRβ in the presence of estrogen (Fig. 6d). This data clearly shows that ERα and ERRβ could bind directly and as ERα/ERRβ heterodimer in the presence of estrogen to regulate ERRβ transcriptionally.

### ERα up-regulates the promoter activity of *ERRβ*

**Fig. 7:**
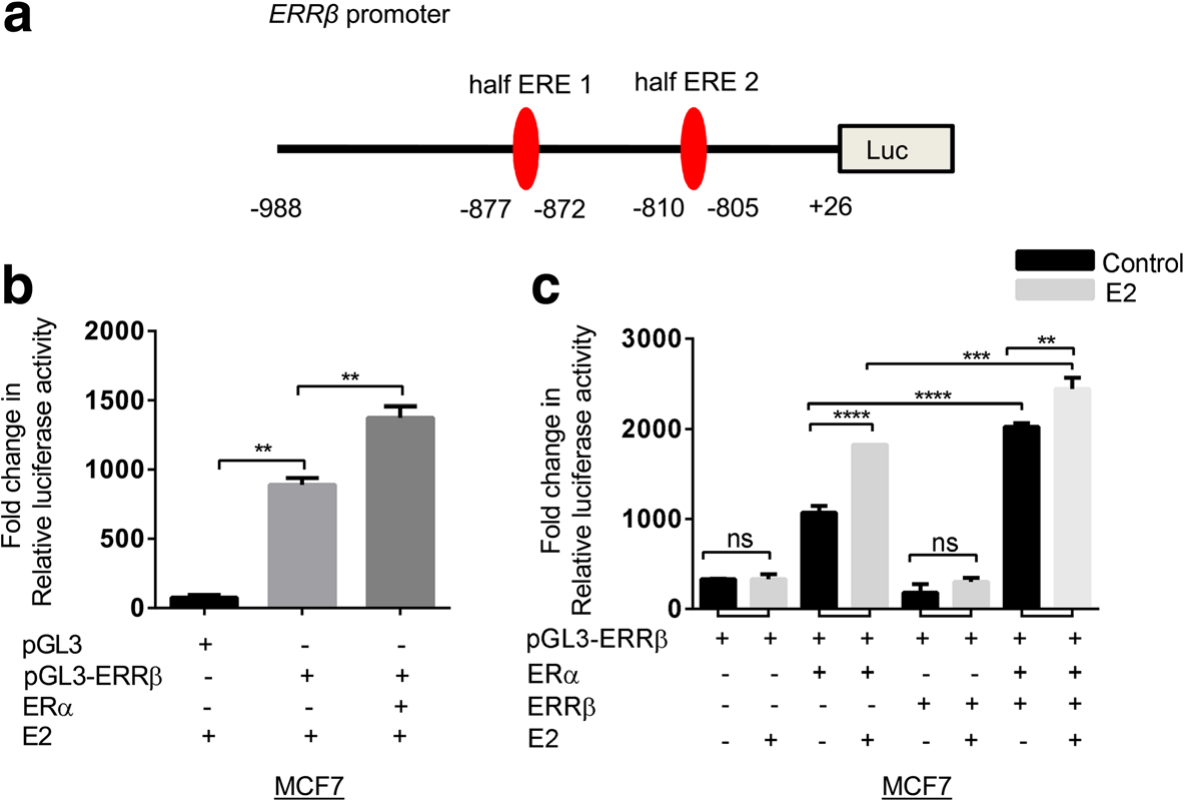
Effect of ERα and ERRβ on *ERRβ* promoter. **a** Schematic representation of *ERRβ* promoter showing two half ERE sites. **b** ERα regulates ERRβ classically in the presence of estrogen. **c** MCF7 cells were transfected with *ERα*, *ERRβ* along with *ERRβ* promoter and luciferase readings were obtained in the presence and absence of estrogen stimulation. Renilla readings were taken as a control and all the experiments were conducted in triplicates; statistical significance was analyzed using One-way ANOVA test and *p* < 0.05 considered as significant (ns- no significance, *p < 0.05, **p < 0.01, ***p < 0.001, ****p < 0.0001)

To further confirm the effect of ERα on *ERRβ* promoter activity, we cloned the *ERRβ* promoter in pGL3 basic luciferase vector using Kpn1 and Xho1 restriction sites. The *ERRβ* promoter construct was sequenced and cloning was confirmed (Fig. 7a). pGL3-*ERRβ* promoter construct was co-transfected with ERα and ERRβ expression vector plasmid. After 48 h of co-transfection with ERα, a significant increase in luciferase activity of *ERRβ* promoter was found (Fig. 7b). The luciferase activity was further elevated in the presence of *ERα* and *ERRβ* followed by estrogen treatment compared to only *ERα* and *ERRβ* co-transfection. However, no significant change was observed in the luciferase activity in the presence of *ERRβ* transfection alone (Fig. 7c). These findings suggest that ERα binds to half ERE sites in the promoter of *ERRβ* to increase its transcription. Apart from that our results also show that ERRβ along with ERα bind to the half ERE sites present on promoter of *ERRβ* gene.

### ERRβ regulates cell cycle in breast cancer cells

**Fig. 8:**
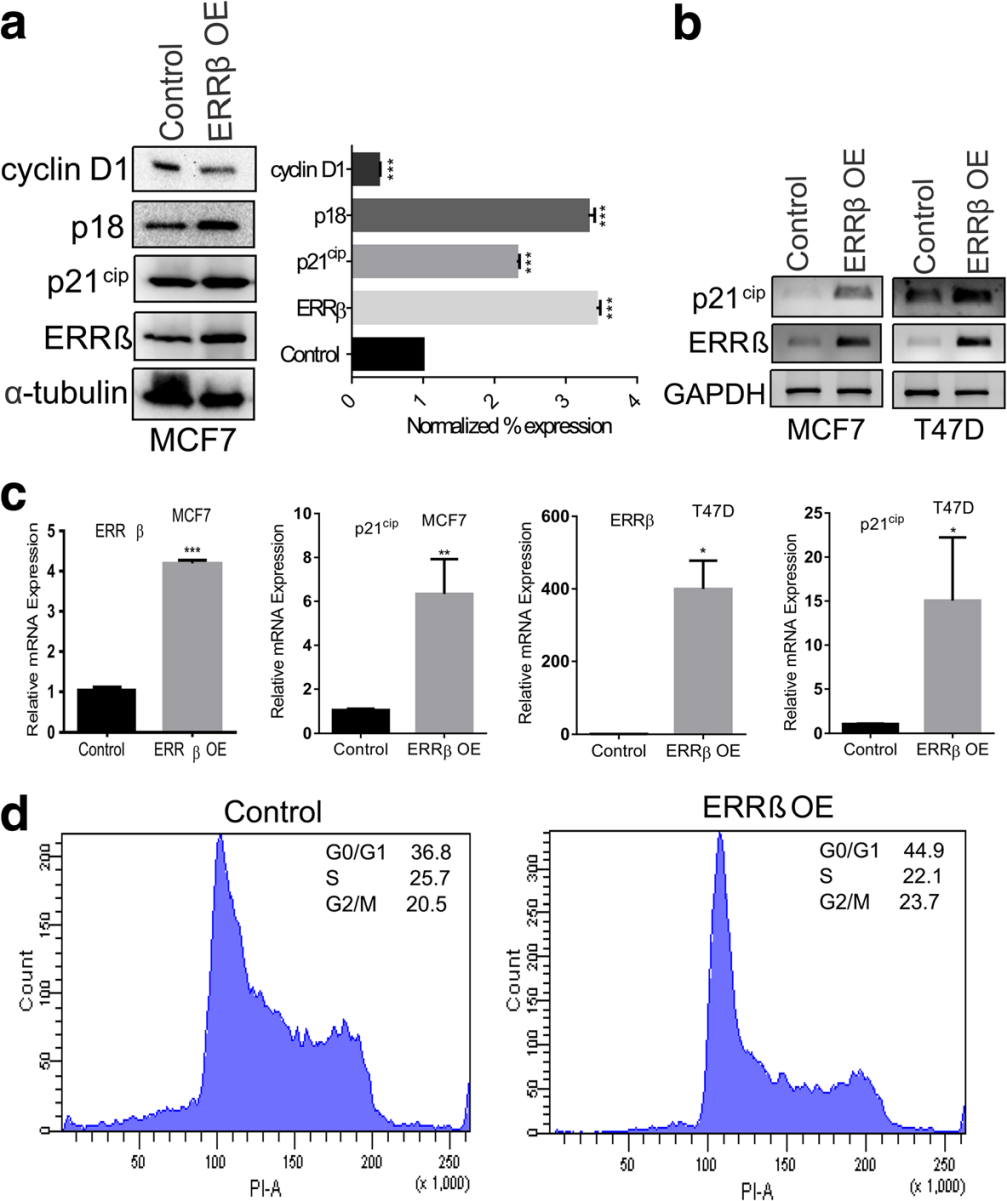
ERRβ is a regulator of cell cycle and inhibition of ERRβ leads to cell proliferation. **a** Western blots and densitometry analysis showing changes in the expression of cell cycle markers, such as p21^cip^, p18 and cyclin D1 upon the over expression of *ERRβ* in MCF7 cells. **b**, **c** *ERRβ* was ectopically expressed in ER + ve breast cancer cells, after 48 h the mRNA levels of p21^cip^ were examined by RT-PCR and RT-qPCR, p21^cip^ was significantly up-regulated. All the results were obtained from three independent experiments and each done in triplicates, 2-group unpaired t-test was used to obtain *p*-values and *p* < 0.05 considered as significant (*p < 0.05, **p < 0.01, ***p < 0.001, ****p < 0.0001)**. d** Fluorescence- activated cell sorting assay (FACS) showing increase of cell fractions in G0/G1 phase upon ectopic expression of ERRβ in MCF7 cells

**Fig. 9:**
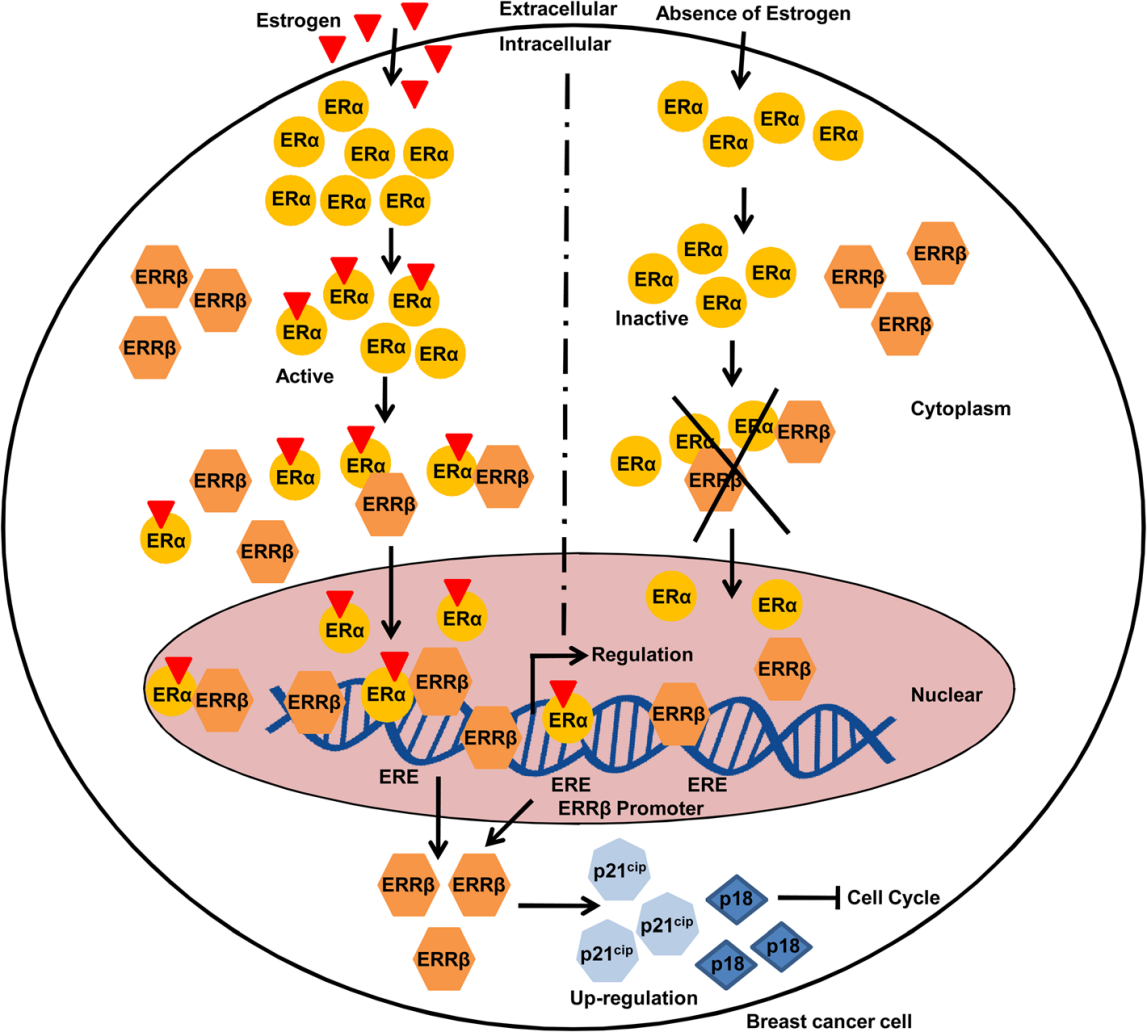
Schematic representation of ERα classically regulating *ERRβ* and role of ERRβ in cell cycle regulation through p18 and p21^cip^. In the presence of estrogen ERα gets activated and forms a heterodimer by interacting with ERRβ. ERα alone or along with ERRβ in the form of heterodimer binds to the promoter region of ERRβ and up-regulates its promoter activity. ERRβ up-regulates cell cycle markers expression such as p21^cip^ and p18. p21^cip^, p18 are cyclin dependent kinase inhibitors and halt cell cycle. Thus, our study suggests that the cell cycle regulating role of ERRβ by up-regulating p21^cip^ and p18

In our present study we demonstrate that ERα can regulate *ERRβ* expression. It has been proven that ERRs share target genes with ERs and p21^cip^ is a target gene of ERα and it has significant role in cell cycle regulation [20, 21, 49]. Hence we hypothesize that ERRβ may also regulate p21^cip^ and has a significant role in cell cycle regulation. To understand the role of ERRβ in cell cycle, we overexpressed *ERRβ* in ER + ve breast cancer cells (MCF7 and T47D). Forty-eight hours of post transfection, the whole cell lysates were extracted from the ERRβ expression vector and control vector transfected cells and western blot was performed. Western blot analyses showed that cell cycle proteins p18 and p21^cip^ were up-regulated whereas cyclin D1 was down-regulated (Fig. 8a). Similar results were also observed in the p21^cip^ mRNA levels in both MCF7 and T47D cells (Fig. 8b, c). These results suggest the probable role of ERRβ in the regulation of cell cycle by regulating p18, p21^cip^ and cyclin D1 in breast cancer cells. Furthermore, fluorescence-activated cell sorting analysis for cell cycle showed increase in G0/G1 phase cell population in ERRβ ectopically expressed cells as expected (Fig. 8d). These results proved the cell cycle regulatory and tumor suppressive role of ERRβ in breast cancer cells. The schematic representation provides an overall idea of the regulation of ERRβ and its role in cell cycle regulation in breast cancer cell lines (Fig. 9).

## Discussion

ERα plays an important role in breast cancer progression, metastasis and treatment [50, 51]. DNA binding domain of ERα is highly conserved with ERRs hence can share target genes [21, 22]. ERRs involve in cell proliferation and energy metabolism [21, 29]. Expression of ERRβ was found to be constant throughout the menstrual cycle [52]. ERRβ can regulate Nanog expression through interacting with Oct4 [53] and acts as tumor suppressor in prostate cancer cells [36]. A limited literature has addressed the role of ERRβ in breast cancer. We therefore studied the possible role of ERRβ in breast cancer. We found the relative expression of ERRβ is high in immortalized normal breast cells (MCF10A), in contrast to breast cancer cell lines (MCF7, T47D, MDA-MB231) and these findings were in agreement with the previous studies [37]. Immunohistochemical staining with ERRβ showed a significant increased expression of ERRβ in normal breast tissues compared to breast carcinoma tissues. Breast cancer patients having high expression of ERRβ showed better survival [47]. Both Immunohistochemical and western blot studies revealed high expression of ERRβ in ER + ve breast cancers and it is dependent on Estrogen receptor status. Furthermore, reduced ERRβ expression was observed in ERα depleted MCF7 cells. These results indicate the possible role of ERα in the regulation of ERRβ in breast cancer. Estrogen is required for the development of breast and ovaries in mammals [54], acts as a ligand for ERs [55], promotes cell proliferation and migration [56]. In our study we attributed the role of estrogen in the regulation of ERRβ in breast cancer cells. We confirmed that the expression of ERRβ is highly elevated in the presence of estrogen in ER + ve breast cancer cells (MCF7). However, in competition studies ERRβ expression was reduced with tamoxifen treatment along with estrogen.

Since ERs and ERRs show sequence similarity, there is a possibility of sharing of target genes and cross-talk between these receptors. In this study we detected two half ERE sites in the upstream region of *ERRβ* and proved the binding of ERα on those ERE sites both in-vitro and in-vivo. ERα interacts with various proteins such as Sp1 and Ap1 which can facilitate the binding of ERα on half ERE sites [57]. Sp1 stabilizes ERα dimer and co-operate the binding of ERα on half EREs present on its target gene promoter [58, 59]. Whereas, HMG1 interacts with ERα and stabilizes ERα-ERE binding through which it enhances the transcription activity [60]. Since previous studies have suggested that ERα is an interacting partner of ERRβ [48], therefore we hypothesize that ERRβ might be playing an important role in the regulation of its own promoter by acting as facilitator of ERα to bind to the half ERE sites. ChIP assay and Re-ChIP provided enough evidenceses to confirm the self regulation of *ERRβ* through ERα in the presence of estrogen. Furthermore, luciferase assay confirmed the regulation of *ERRβ* by ERα. Surprisingly, *ERRβ* alone has no effect on promoter activity. These findings demonstrate that ERα can regulate the transcriptional activity of *ERRβ*.

In normal cells the cell division is tightly regulated and a fine balance amongst the cell cycle modulators does exist [61]. The impairment of this fine balance is one of the major causes of cancer. p21^cip^ is an inhibitor of cyclin dependent kinase belongs to cip and kip family [62], primarily inhibits CDK2 by which it can inhibit cell cycle progression [63, 64]. p21^cip^ arrests G1-G2 transition in cell cycle through binding to PCNA in P53 deficient cells [65]. p18 belongs to INK4 family and can inhibit cyclin dependent kinases potentially. Reduced levels of p18 were detected in hepatocellular carcinoma [66]. In this study, we have established the correlation between the expression of ERRβ and various cell cycle markers such as p21^cip^, p18 and cyclin D1 in breast cancer cells. The elevated levels of p21^cip^, p18 and decreased expression of cyclin D1 in ectopically expressed ERRβ breast cancer cell lines were observed. Cell cycle analysis (FACS) provided enough evidence of cell cycle regulatory role of ERRβ in MCF7 cells. p21^cip^ protein levels were directly correlated with the expression of ERRβ in prostate cancer cells and it has been proved that p21^cip^ is a direct target for ERRβ [36]. Interestingly p21^cip^ was demonstrated as a direct target for both ERRα and ERRγ and their protein levels were negatively correlated with each other [67, 68]. Thus, not only for ERRβ, p21^cip^ is a direct target for all ERRs. Prostate and breast cancer cells showed inhibition of ERRα using XCT790 (inverse agonist) leads to reduction in cell proliferation [67]. However, ERRβ and ERRγ were served as tumor suppressors in prostate cancer cells [36, 68]. Recent studies also demonstrated the tumor suppressor role of ERRβ through BCAS2 in breast cancer cells [37]. Our results were in agreement with the previous studies and this cell cycle regulatory and tumor suppressor roles of ERRβ in breast cancer cells suggest that ERRβ can be considered as a potential therapeutic target for the treatment of breast cancer.

One might surprise with the tumor suppressive role of an estrogen induced gene. It is well established that estrogen promotes cell proliferation in ER + ve breast cancer cells but also induces the expression of p53 and BRCA1. Interestingly, not only p53 but also BRCA1 gene is associated with inhibition of cell growth, DNA repair and apoptosis [69–73]. P53 and BRCA1 both physically interact with ERα and inhibit ERα-mediated transactivation [74, 75]. Recent studies also showed that estrogen up-regulate the expression of RERG a novel tumor suppressive gene which is highly expressed in ER + ve breast cancers [76]. In our study we showed that ERRβ is an estrogen responsive gene and it exhibits tumor suppressor role in breast cancer cells. Recent studies showed that ERRβ interacts with ERα in the presence of estrogen and ERRβ decrease the intranuclear mobility through which it can inhibit the transcriptional activity of ERα [48]. This phenomenon might be playing an important role in the inhibition of estrogen responsive target genes. Hormonal activation of tumor suppressive genes such as p53, BRCA1, RERG and ERRβ do play a vital role in the regulatory pathways that inhibit the estrogen induced cell growth and differentiation.

## Conclusions

In our present study we have categorically demonstrated that ERRβ expression was down-regulated in the breast cancer patient samples in comparison with normal samples. High expression of ERRβ showed a significant favorable survival outcome in breast cancer. We showed for the first time that the expression of ERRβ is ERα dependent and stimulated by steroid hormone estrogen as observed in patient data and breast cancer cell lines. In vitro and In vivo studies proved that ERRβ is a direct target of ERα. Cyclin D1, p21^cip^ and p18 plays an important role in cell cycle and we also have established the correlation between the expression of ERRβ and the expression of these cell cycle modulators. Therefore our study proposes that ERRβ could be a possible tumor suppressor and can be used as therapeutic target in breast cancer.

## Abbreviations

BC: Breast cancer
ChIP: Chromatin immunoprecipitation
DMEM: Dulbecco’s modified Eagle’s medium
E2: Estrogen
EMSA: Electrophoretic mobility shift assay
ER: Estrogen receptor
ERR: Estrogen related receptor
FBS: Fetal bovine serum
GAPDH: Glyceraldehyde-3- phosphate dehydrogenase
qRT-PCR: Quantitative real-time polymerase chain reaction
RPMI 1640: Roswell Park Memorial Institute 1640
RT-PCR: Reverse transcription polymerase chain reaction
shRNA: Short hairpin RNA
TMA: Tissue microarray
